# Revisiting Ceriantharian (Anthozoa) Mitochondrial Genomes: Casting Doubts about Their Structure and Size

**DOI:** 10.1093/gbe/evaa130

**Published:** 2020-06-26

**Authors:** David Roy Smith

**Affiliations:** Department of Biology, University of Western Ontario, London, Ontario, Canada

**Keywords:** Ceriantharia, Cnidaria, *Isarachnanthus*, linear mitochondrial genome, *Pachycerianthus*

## Abstract

Recently, Stampar et al. (2019. Linear mitochondrial genome in Anthozoa (Cnidaria): a case study in. Sci Rep. 9(1):6094.) uncovered highly atypical mitochondrial genome structures in the cnidarian species *Pachycerianthus magnus* and *Isarachnanthus nocturnus* (Anthozoa, Ceriantharia). These two mitochondrial DNAs assembled as linear fragmented genomes, comprising eight and five chromosomes, respectively—architectures unlike any other anthozoan mitogenome described to date. What’s more, they have cumulative lengths of 77.8 (*P. magnus*) and 80.9 kb (*I. nocturnus*), making them the largest animal mitochondrial DNAs on record, a finding which garnered significant attention by various news media. Here, I take a closer look at the work of Stampar et al. and question their key results. I provide evidence that the currently available mitogenome sequences for *I. nocturnus* and *P. magnus*, including their structures, sizes, and chromosome numbers, should be treated with caution. More work must be done on these genomes before one can say with any certainty that they are linear, fragmented, or the largest animal mitogenomes observed to date.

**Significance**

A recent study by Stampar et al. (2019. Linear mitochondrial genome in Anthozoa (Cnidaria): a case study in. Sci Rep. 9(1):6094.) described a linear fragmented mitochondrial genome architecture in two species from the anthozoan order Ceriantharia (“tube anemones”). Here, I revisit the data of Stampar et al. (2019) and question their key results, arguing that the described structures, sizes, and chromosome numbers of these two mitochondrial DNAs (mtDNAs) should be treated with caution.

## Introduction

These days, it is rare to come across a truly unusual mitochondrial genome, especially one from an animal. Indeed, more than 9,500 animal mtDNAs have been sequenced, covering almost every branch of the metazoan tree of life and encompassing a marvelous array of genomic architectures (Smith and Keeling 2015; Lavrov and Pett 2016). That is why a study by Stampar et al. (2019), describing the atypical mtDNAs of two cnidarian species, grabbed my attention, but has also led me to question some of their findings.

The phylum Cnidaria, which includes corals, jellyfish, and sea anemones, is a hotspot for unconventional mtDNAs (Kayal et al. 2012; Lavrov and Pett 2016; Yahalomi et al. 2020). For example, the mitogenome of the winged box jellyfish (Medusozoa, Cubozoa) is fragmented into eight linear chromosomes, each containing one to five genes as well as defined telomeres (Smith et al. 2012). In fact, linear or linear fragmented mtDNA appears to be a universal theme throughout the Medusozoa (Kayal et al. 2012), whereas the Anthozoa—the other major cnidarian lineage (Kayal et al. 2018)—is characterized by having circular-mapping mtDNA (Lavrov and Pett 2016). However, no complete mitogenomes have been reported for the anthozoan order Ceriantharia (“tube anemones”). That is, until the recent work of Stampar et al. (2019), which presented the mtDNAs of the ceriantharians *Pachycerianthus magnus* and *Isarachnanthus nocturnus*. Their findings were quite remarkable.

The *P. magnus* and *I. nocturnus* mtDNAs assembled as linear fragmented genomes, comprising eight and five chromosomes, respectively—architectures unlike any other anthozoan mitogenome described to date. What’s more, these two mtDNAs have cumulative lengths of 77.8 (*P. magnus*) and 80.9 kb (*I. nocturnus*), which, if correct, makes them the largest animal mtDNAs on record. As one might expect, these data have garnered significant attention online, including a recommendation in *F1000Prime*, and coverage by various news media, such as *EurekAlert*, which ran the headline: “Simple sea anemones not so simple after all.”

## Digging Up the Sequence Data

My concerns began when I tried to access the *P. magnus* and *I. nocturnus* mtDNA sequences. The authors write that the “data are available via GenBank … as SAMN10291198 (*I. nocturnus*) and SAMN10291199 (*P. magnus*)” (Stampar et al. 2019). Following these accessions at the National Center for Biotechnology Information (NCBI) website takes you to a BioSample entry for each of the species, which lists details about the specimens used for mitogenome sequencing (e.g., origin of isolation) as well as a link to a BioProject. Under the BioProject, there are two Sequence Read Archive entries (discussed below) but no links to nucleotide sequence accessions for the assembled mitochondrial genomes.

Consequently, I downloaded all the available nucleotide sequences for *I. nocturnus* and *P. magnus* from GenBank and, still, I could not find the mtDNA chromosomes, nor were they present in any of the Organelle Genome Resources databases. Even BLAST searches using previously published mtDNA genes from these species (GenBank accessions AB859841 and JX128342) did not recover the genomes. Disappointed that I could not get annotated chromosomes for these interesting mitogenomes, I moved on to the Sequence Read Archive (SRA) entries for *I. nocturnus* and *P. magnus*, and that’s when things got confusing.

The *I. nocturnus* and *P. magnus* mtDNAs were sequenced on an Illumina HiSeq 2500 platform, yielding 14.2 and 15.3 million paired-end reads, respectively (Stampar et al. 2019). However, when I downloaded the SRA entries for these species (SRX4936394 and SRX4936395), I had a great surprise: the files did not contain Illumina sequencing reads, they contained the assembled mitochondrial genomes! It did not take me long to figure this out as there were five “Illumina” sequences for *I. nocturnus*, each with a length that matched that of one of the described mitochondrial chromosomes. For *P. magnus*, things were a bit more confusing as only seven of the eight mitochondrial chromosomes were present in the SRA file (the longest chromosome was missing).

This is problematic for a few reasons, not to mention that the SRA is technically reserved for sequencing reads, not assembled genomes. First, it means that no annotations are available for these genomes, and in one case a whole chromosome is missing. Second, the data are not easily identified through the normal means, such as BLAST searches or through NCBI’s Refseq release of mitochondrial sequences. Third, when downloading the SRA files through the NCBI website, all sequences get cleaved to a length of 10 kb or less, resulting in a loss of data for any mtDNA chromosome that is >10 kb. Finally, substituting the genomes for the Illumina data means that the raw sequencing reads are not available, but I would argue that they are the most crucial component of the study—the very data on which the key conclusions are based. Note: I emailed the corresponding (Stampar) and senior (Daly) authors of the study asking them for access to the reads, explaining that I wanted to reanalyze and re-evaluate the assembly and architecture of the *P. magnus* and *I. nocturnus* mtDNAs. They were not forthcoming with the data, noting that the reads are being used for ongoing analyses and collaborations, and expressing concerns about being scooped on these future projects.

## Are the mtDNAs Linear and Fragmented?

Proving that an organelle genome is linear is no easy task. Stampar et al. (2019) argued throughout their paper that the *I. nocturnus* and *P. magnus* mitochondrial chromosomes are linear, but their only evidence for this is based on the de novo assembly of Illumina data (read length = 250 nt), followed by subsequent rounds of mapping reads to reference sequences. In short, the mtDNA-derived contigs that they identified from the de novo assembly did not have circular maps (but see evidence contradicting this below), and all attempts to extend the contigs by read mapping failed. However, there are other reasons why reads may not extend beyond the ends of a given contig even when that contig comes from a circular-mapping chromosome. Illumina sequencing is a finicky process and many factors can lead to a sudden drop in coverage and/or sequence-specific errors (Nakamura et al. 2011; Ekblom et al. 2014), including repeats and/or sequences that readily form secondary structures.

What is most conspicuous about the characterization of the *I. nocturnus* and *P. magnus* mtDNAs is what’s missing. No restriction digests, gel-electrophoresis, or southern blots were carried out to confirm a linear architecture. There is no mention of any PCR experiments to try and bridge the ends (either within or between chromosomes). And, apart from looking for inverted repeats within the contigs, no attempts were made to describe the structures of the telomeres. With very few exceptions, linear-mapping organelle genomes have defined telomeric regions, typically (but not always) arranged as inverted repeats and often having complicated arrangements, such as closed single-stranded loops (Nosek et al. 1998). At present, not one defining telomeric feature has been identified in the mtDNAs of *I. nocturnus* and *P. magnus*.

Some of the presented data actually contradict a linear—or at least a linear fragmented—structure. For instance, Stampar et al. (2019) found that “a small percentage of the paired-end reads mapped across the possible chromosomes: 1,238 PE reads (1%) for *P. magnus* and 1,341 PE reads (0.5%) for *I. nocturnus*,” which they reasoned “is likely an artifact of the mapping due to their relatively low occurrence.” Still, we are talking about thousands of reads that bridged different chromosomes. In the very least, such a finding should warrant PCR analysis to see if the mappings are in fact artifacts. A common theme among linear organelle DNA assemblies is a sharp drop in read coverage at the ends of the chromosome (Voigt et al. 2008; Janouškovec et al. 2013; Hamaji et al. 2017). Although the Illumina reads used for assembling the *I. nocturnus* and *P. magnus* mtDNAs are unavailable, the authors do provide a map of the read coverage across the chromosomes in the Supplementary Material (Stampar et al. 2019) . Looking closely at this figure, one can see a sharp spike in read coverage toward the ends of many of the chromosomes, which in my opinion does not support a linear structure. Moreover, this supplementary figure also shows nine mitochondrial chromosomes for *P. magnus* (mislabeled *I. nocturnus*), instead of the eight described in the main text. In addition, in organisms with partitioned mitochondrial chromosomes, there are often differences in copy number between the different chromosomes (Suga et al., 2008; Wei et al., 2012). The fact that the coverage of the regions encoding protein-coding genes is very similar between the putative chromosomes (as presented in the supplementary figure of Stampar et al. (2019)) could be an indication that the ceriantharian mtDNA “chromosomes” are in fact contigs of the same chromosome.

**Fig. 1.**
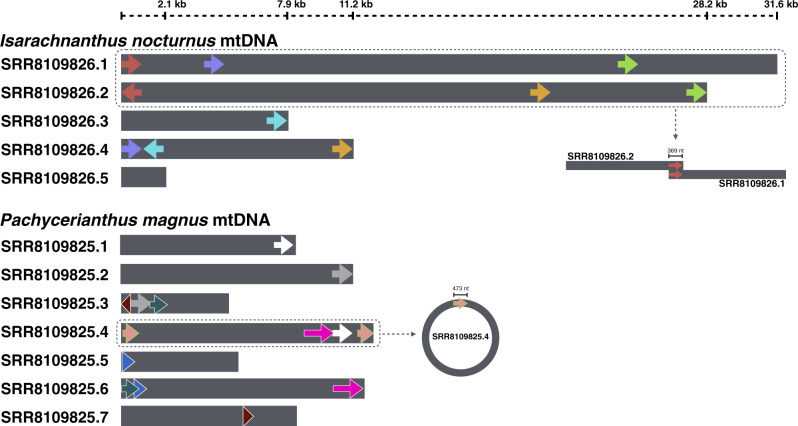
Repeat elements identified in the linear, fragmented mtDNAs of *Pachycerianthus magnus* and *Isarachnanthus nocturnus* from Stampar et al. (2019). Mitochondrial chromosomes are drawn to scale (SRA accession numbers are shown). Repeats (not drawn to scale) are shown with colored arrows (same color, identical repeat). All repeats are between 100 and 500 nt. This figure highlights only a small proportion of the repeats identified within these genomes. Dashed boxes and lines denote contigs that can easily be assembled together or folded into a circular molecule.

To get a better understanding of the *I. nocturnus* and *P. magnus* mitogenomes, I downloaded their sequences (save for *P. magnus* chromosome 1) and blasted each mtDNA against itself—in other words, I blasted the individual chromosomes against each other (BlastN implemented through Geneious v10.2.6., Biomatters Ltd., Auckland, New Zealand, using default settings). The results from this simple experiment uncovered a moderate number (>25) of long (100–350 nt), highly conserved (>98% identify) repeats within the *I. nocturnus* and *P. magnus* mitochondrial genomes, which were not discussed in any detail in the paper. In many instances, >250 nt from the extreme ends of chromosomes match with 100% identity to segments on other chromosomes (fig. 1). For example, the first 369 nt of mitochondrial chromosome 1 from *I. nocturnus* (SRR8109826.1) matched perfectly to the start of chromosome 2 (SRR8109826.2), but in reverse orientation; consequently, these two chromosomes can easily be assembled into a single contig (fig. 1). Likewise, nucleotides 4–476 of *P. magnus* mtDNA chromosome 4 share 100% sequence identity with the last 473 nt of this chromosome (11,586–12,058), meaning it can be folded into a circular molecule (fig. 1). This is significant because a fragmented circular mtDNA (2 chromosomes) was recently identified in the anthozoan *Umbellula* (Hogan et al. 2019).

I am reluctant to derive too much meaning from these BLAST analyses, but I believe that the identified repeat elements could have resulted in—or are evidence of—assembly errors, especially when considering that these repeats can be 100% identical and longer than the Illumina reads used to assemble the genomes. The repeats could also be a sign of a more complicated underlying genomic architecture (Lavrov et al. 2016). Moving forward, I would argue that the currently available mitogenome sequences for *I. nocturnus* and *P. magnus*, including their structures, sizes, and chromosome numbers, need to be treated with caution. More work must be done on these genomes before one can say with any certainty that they are linear, fragmented, or the largest animal mtDNAs observed to date. Long-read PacBio sequencing would be particularly helpful in resolving the sequences and arrangements of these genomes, as would accompanying restriction digest and gel-electrophoresis experiments.

I should emphasize that I think the work of Stampar et al. (2019) is well written and interesting, and in no way am I suggesting that they manipulated their results or knowingly misled readers—although I would recommend that they revise and update the GenBank data for the *I. nocturnus* and *P. magnus* mtDNAs. Some of my concerns with the paper could have been dealt with through personal communication with the corresponding author. However, given that the study has already been widely disseminated for over 12 months and has the strong potential to be highly cited in the coming years, I thought it best to have these issues documented within the scientific literature. I have no doubt that future work will prove that the *I. nocturnus* and *P. magnus* mtDNAs are interesting—perhaps even stranger than initially thought. But, as it stands, I believe their sizes and structures are undetermined. 
